# Iron regulates the expression of ferroportin 1 in the cultured hFOB 1.19 osteoblast cell line

**DOI:** 10.3892/etm.2014.1823

**Published:** 2014-07-03

**Authors:** GUO-YANG ZHAO, DONG-HUA DI, BO WANG, PENG ZHANG, YOU-JIA XU

**Affiliations:** 1 Department of Orthopedics, The Affiliated Hospital of Jiangsu University, Zhenjiang, Jiangsu 212000, P.R. China; 2 Department of Orthopedics, The Second Affiliated Hospital of Soochow University, Suzhou, Jiangsu 215004, P.R. China

**Keywords:** iron metabolism, ferroportin 1, osteoblast

## Abstract

Iron metabolism is tightly regulated in osteoblasts, and ferroportin 1 (FPN1) is the only identified iron exporter in mammals to date. In the present study, the regulation of FNP1 in human osteoblasts was investigated following various iron treatments. The human osteoblast cell line hFOB 1.19 was treated with ferric ammonium citrate (FAC) or desferrioxamine (DFO) of various concentrations. The intracellular iron ion levels were measured using a confocal laser scanning microscope. In addition, the mRNA and protein expression levels of FPN1 were detected by quantitative polymerase chain reaction, western blot analysis and immunofluorescence. The results demonstrated that increasing iron concentrations via FAC treatment increased the expression of FPN1. By contrast, decreasing the iron concentration by DFO treatment decreased FNP1 expression levels. In addition to demonstrating that the FNP1 expression changed according to the iron concentration, the observations indicated that changes in FPN1 expression may contribute to the maintenance of the intracellular iron balance in osteoblasts.

## Introduction

Iron is one of the most important trace elements found in the human body. As with all cells, bone cells require iron for numerous aspects of their physiology. A number of studies have indicated that bone metabolism is closely associated with iron metabolism. In particular, iron overload and iron deficiency may lead to osteopenia or even osteoporosis (1–3). Iron chelation therapy has been demonstrated to improve osteoporosis in ovariectomized rats (4,5). In addition, hepcidin (a peptide hormone that decreases iron levels in the body) has also been investigated for the treatment of osteoporosis in peri- and post-menopausal females (6). These studies indicate that iron plays an important role in bone metabolism. Therefore, research into the mechanisms underlying the iron balance in bone cells is crucial to improve the understanding of the pathogenesis and treatment of iron-associated bone disease.

*In vitro* studies have previously revealed that iron excess inhibits osteoblastic metabolism due to the damage caused by oxidative stress (7–9), while iron deficiency can inhibit osteoblastogenesis due to the decreased activity of ribonucleotide reductase (10,11). Ferroportin 1 (FPN1), which contributes to iron release from cells and the maintenance of iron homeostasis, is currently the only iron exporter to be identified in mammals (12–14). The exporter is highly expressed in macrophages, enterocytes and hepatocytes (15,16). A recent study demonstrated that FPN1 is also expressed in human osteoblasts (17). The expression of FPN1 in macrophages (18–20), enterocytes (21,22), hepatocytes (23) and cardiocytes (24) has been reported to be regulated by iron concentration; however, the association between FPN1 and iron ion levels in osteoblasts is yet to be fully elucidated. In the present study, the human osteoblast cell line hFOB 1.19 was treated with ferric ammonium citrate (FAC) or desferrioxamine (DFO) of various concentrations. The intracellular levels of iron ions were measured using confocal laser scanning microscopy (CLSM). In addition, the mRNA and protein expression levels of FPN1 were detected by quantitative polymerase chain reaction (qPCR), western blot analysis and immunofluorescence. The aim of the present study was to provide further information to improve the understanding of the role that FPN1 plays in osteoblastic iron metabolism.

## Materials and methods

### Cell cultures and treatments

The hFOB 1.19 cell line (Shanghai Institute of Biochemistry and Cell Biology, Shanghai, China) was maintained in Dulbecco’s modified Eagle’s medium-F_12_, supplemented with 10% fetal bovine serum and 3% G418 disulfate solution, in a humidified atmosphere of 5% CO_2_ in air at 34°C. The medium was replenished every 2–3 days. After reaching 70–80% confluence, the cells were passaged by treatment with 0.05% trypsin. For CLSM, qPCR and western blot analysis, FAC (Sinopharm Chemical Reagent Co. Ltd., Shanghai, China) and DFO (Novartis Pharma Schweiz AG, Rotkreuz, Switzerland) were added to the medium at final concentrations of 50, 100 and 200 μmol/l for FAC as treatment in the iron excess group and 5, 10 and 20 μmol/l for DFO as treatment in the iron deficiency group. For immunofluorescence FPN1 analysis, 50 μmol/l FAC and 10 μmo/l DFO were added for the iron excess and iron deficiency groups, respectively. In the control, the same amount of medium without FAC or DFO was used. Cells were incubated with FAC and DFO for 20 h.

### Confocal microcopy measurements

The hFOB 1.19 cells were seeded on coverslips for the analysis of iron ions by fluorescence quenching. Briefly, following treatment with FAC and DFO for 20 h, the hFOB 1.19 cells were washed twice with phosphate-buffered saline (PBS) and incubated with Phen Green FL (Molecular Probes, Eugene, OR, USA) away from light at 34°C in a humidified atmosphere containing 5% CO_2_ for 30 min. Next, the cells were washed twice with PBS to remove the unbound fluorescent indicator, and then incubated with the culture medium for an additional 15 min. CLSM model TCS-SP2 (Leica, Wetzlar, Germany) was used to measure the green fluorescence of Phen Green FL when excited at 488 nm and emitted at 521 nm.

### qPCR analysis

Total RNA was extracted from the hFOB 1.19 cells following treatment using TRIzol reagent (Invitrogen Life Technologies, Carlsbad, CA, USA) and single-stranded cDNA was synthesized using a reverse transcription kit purchased from Promega Corporation (Madison, WI, USA), according to the manufacturer’s instructions. qPCR was performed using a real-time PCR system (Applied Biosystems Step One; Thermo Fisher Scientific, Waltham, MA, USA). Amplification reactions were conducted in a 20-μl volume using SYBR-Green I dye under the following amplification conditions: 30 cycles of 94°C for 30 sec, 50°C for 30 sec and 72°C for 30 sec. Primers were designed to specifically amplify 176 bp of human FPN1 cDNA (forward, 5′-CTACTTGGGGAGATCGGATGT-3′ and reverse, 5′-CTGGGCCACTTTAAGTCTAGC-3′); and 306 bp of human β-actin cDNA (forward, 5′-TCCTGTGGCATC CACGAAACT-3′ and reverse, 5′-GAAGCATTTGCGGTG GACGAT-3′). The mRNA/cDNA abundance of each gene was calculated relative to the expression of the housekeeping gene, β-actin. Relative quantification was calculated using the 2^−ΔΔCT^ method and analysis was performed using Step One™ Software V 2.1 (Thermo Fisher Scientific).

### Western blot analysis

Total protein was extracted from the hFOB 1.19 cells following treatment using radioimmunoprecipitation assay buffer, and separated on 6% SDS gel prior to transfer onto polyvinylidene difluoride membranes. The membranes were blocked in 5% (m/v) milk dissolved in Tris-buffered saline with 0.05% (w/v) Tween-20 (TBS-T) and incubated overnight at 4°C with rabbit anti-ferroportin, (1:200) or anti-β-actin (1:500; Abcam, Cambridge, MA, USA) primary antibodies. Following washing three times with TBS-T at room temperature, the membranes were incubated for 1 h with goat peroxidase-labeled anti-rabbit immunoglobulin (1:500), and visualized with enhanced chemiluminescence (Amersham Biosciences Corporation, Piscataway, NJ, USA). The images were analyzed with ImageJ software (National Institutes of Health, Bethesda, MD, USA).

### Immunofluorescence analysis

Cells were seeded on glass coverslips for immunofluorescence analysis. Following treatment, the cells were fixed with 4% paraformaldehyde for 15 min and washed twice with PBS. The cells were then incubated in a blocking solution (5% bovine serum albumin) for 30 min at room temperature, followed by incubation with primary antibodies (rabbit anti-ferroportin 1; 1:50; Abcam) in a humid chamber at 4°C overnight. Following washing three times with PBS, the cells were incubated with a fluorescein isothiocyanate-conjugated goat anti-rabbit antibody (1:1,000; Jackson ImmunoResearch Laboratories, Inc., West Grove, PA, USA) at room temperature for 30 min. Coverslips were then washed three times with PBS, mounted and observed using a fluorescent microscope (Axio Observer A1; Carl Zeiss AG, Oberkochen, Germany).

### Statistical analysis

Data are expressed as the mean ± standard deviation, and were analyzed with one-way analysis of variance with post-hoc analysis using SPSS version 15.01 for Windows (SPSS, Inc., Chicago, IL, USA). P<0.05 was considered to indicate a statistically significant difference.

## Results

### Intracellular fluorescence quenching by iron following treatment with FAC and DFO

**Figure 1:**
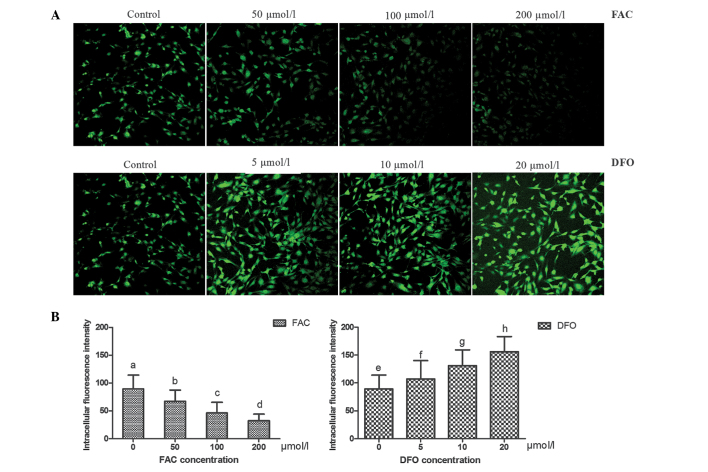
Confocal microscopy analysis of the iron concentration in osteoblasts. (A) The quenching of the fluorescence of Phen Green FL-labeled cells by iron ions was measured with a confocal laser-scanning microscope. Representative microscopic fields are shown (magnification, ×20). (B) A correlation between fluorescence intensity and intracellular iron concentration was observed; the fluorescence intensity significantly weakened with increasing FAC concentrations, but was enhanced with increasing of DFO concentrations. Results are expressed as the mean ± standard deviation of three independent experiments. Means with different letters are significantly different (P<0.05). FAC, ferric ammonium citrate; DFO, desferrioxamine. a–h indicate that all the values in each bar graph are statistically significantly different.

The hFOB 1.19 cells in culture exhibited typical spindle and polygon shapes. Following exposure to various concentrations of FAC and DFO for 20 h, a correlation between the fluorescence intensity in the hFOB 1.19 cells and the intracellular iron concentration was observed; the fluorescence intensity significantly weakened with increasing FAC concentrations, but was enhanced with increasing DFO concentrations (P<0.05 for all comparisons; Fig. 1). These observations indicate that FAC effectually increased the intracellular iron concentration, while DFO effectually decreased the intracellular iron levels.

### mRNA and protein expression levels of FPN1 following treatment with FAC and DFO

**Figure 2:**
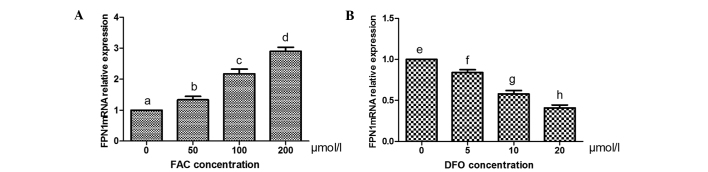
Effect of excessive iron and iron deficiency on the mRNA expression of FPN1 in osteoblasts. (A) RNA expression levels of FPN1 in osteoblasts increased with increasing FAC concentrations in a concentration-dependent manner. (B) mRNA expression levels decreased with increasing DFO concentrations in a concentration-dependent manner. Results are expressed as the mean ± standard deviation of three independent experiments. Means with different letters are significantly different (P<0.05). FAC, ferric ammonium citrate; DFO, desferrioxamine; FPN1, ferroportin 1. a–h indicate that all the values in each bar graph are statistically significantly different.

**Figure 3:**
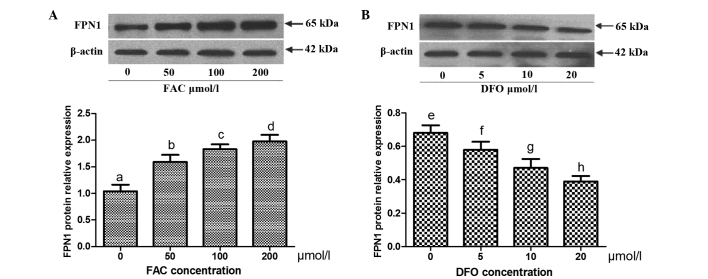
Effect of excessive iron and iron deficiency on the protein expression of FPN1 in osteoblasts. Representative agarose gel images and quantitative analyses showing the protein expression levels of FPN1 in cells treated with (A) FAC and (B) DF. FPN1 protein expression increased with increasing FAC concentrations in a concentration-dependent manner, and decreased with increasing DFO concentrations in a concentration-dependent manner. Results are expressed as the mean ± standard deviation of three independent experiments. Means with different letters are significantly different (P<0.05). FAC, ferric ammonium citrate; DFO, desferrioxamine; FPN1, ferroportin 1. a–h indicate that all the values in each bar graph are statistically significantly different.

**Figure 4:**
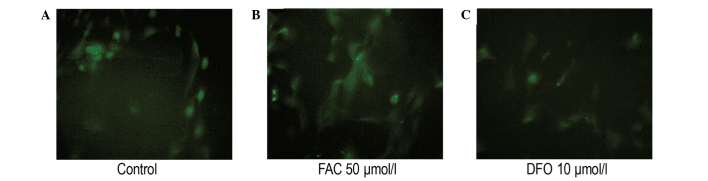
Immunofluorescence analysis of FPN1 protein expression in osteoblasts. When compared with the (A) control group, the intensity of FPN1 fluorescence in cells treated with (B) 50 μmol/l FAC was significantly increased, while the fluorescence intensity in cells treated with (C) 10 μmol/l DFO was significantly decreased (magnification, ×40). FAC, ferric ammonium citrate; DFO, desferrioxamine; FPN1, ferroportin 1.

qPCR revealed that the mRNA expression levels of FPN1 in osteoblasts increased with increasing concentrations of FAC in a concentration-dependent manner, whereas they decreased with increasing concentrations of DFO in a concentration-dependent manner (P<0.05 for all comparisons; Fig. 2). Western blot analysis demonstrated the same pattern of FPN1 expression at the protein level (Fig. 3). Immunofluorescence analysis revealed that the intensity of FPN1 fluorescence in the cells treated with 50 μmol/l FAC was significantly increased when compared with that of the control. In addition, the fluorescence intensity in the cells treated with 10 μmol/l DFO was significantly decreased when compared with that of the control (Fig. 4).

## Discussion

Previous studies have demonstrated that the expression of intracellular FPN1 may be regulated by iron levels in a number of cell types. In 2002, Yang *et al* (25) found that the mRNA expression of FPN1 was significantly increased in human lung macrophages treated with excessive iron using *in situ* hybridization. In 2003, Knutson *et al* (18) reported that iron excess increased the mRNA expression levels of FPN1, while iron deficiency decreased the mRNA expression levels of FPN1 in murine J744 macrophages. Furthermore, this effect was completely inhibited by actinomycin D, an inhibitor of RNA polymerase. The results of these studies indicated that the regulation of FPN1 by iron occurs at a transcriptional level in macrophages. In the present study, iron ions were added to the medium in the form of FAC, and a chelator of iron ions was added in the form of DFO. The iron content of the treated cells was measured by CLSM and the results confirmed that iron excess or iron deficiency was achieved in osteoblasts treated with FAC or DFO, respectively. Iron excess was shown to increase the mRNA expression of FPN1 in osteoblasts, while iron deficiency decreased the mRNA expression of FPN1 in osteoblasts. These observations indicated that the regulation of FPN1 by iron also occurs at a transcriptional level in osteoblasts. The mechanism underlying regulation at a transcriptional level has been demonstrated to be associated with nuclear factor erythroid-derived 2-like 2 (Nrf2), a transcriptional activator (26). In the case of iron excess, oxidative stress was increased, resulting in Nrf2 nuclear accumulation and the promotion of FPN1 mRNA transcription. In the case of iron deficiency, oxidative stress was decreased, leading to a reduction in Nrf2 expression, thereby the transcription of FPN1 mRNA was inhibited (27).

In recent years, a number of studies have demonstrated that the 5′-untranslated region (UTR) of FPN1 mRNA contains an iron responsive element (IRE). This structure indicates that the regulation of FPN1 expression may occur at a translational level, in a similar manner to ferritin and mitochondrial aconitase (28,29). The presence of the IRE in the 5′-UTR of the mRNA functions as a negative regulator of translation. When intracellular iron levels are high, the activity levels of iron regulatory proteins (IRPs) decrease and, thus, are unable to bind to the 5′-IRE, leading to increased translation of FPN1 mRNA and a release of iron. By contrast, when intracellular iron levels are low, IRPs bind to the 5′-IRE and inhibit the translation of FPN1 mRNA, which results in the decreased release of iron (30). The IRE of FPN1 mRNA has been shown to be functional in a variety of cell types, including the human monocytic cell line U937 (31), and the mouse macrophage cell line RAW264.7 (32). Furthermore, the expression of FPN1 has been shown to be unaffected by iron treatment in HepG2 and Caco-2 cells with knockout IRE (31). The present study demonstrated that with changes to the intracellular iron content, the expression of PFN1 at the protein level also changes. Therefore, we hypothesized that control of the level of translation may also be involved in the regulation of FPN1 expression in osteoblasts. The regulation of FPN1 at a transcriptional and translational level may be conductive to the balance of intracellular iron. In addition, this process is useful in decreasing iron-mediated oxidative stress to osteoblasts.

In the present study, excessive iron was shown to increase the expression of FPN1, while iron deficiency decreased the expression of FPN1 in osteoblasts. Similar results have also been observed in bronchial epithelial cells (25), macrophages (18–20), cardiocytes (24) and hepatocytes (23). However, the expression of FPN1 exhibited opposite effects in enterocytes (21,22) and placental syncytiotrophoblast cells (33), where excessive iron decreased the expression of FPN1 and iron deficiency increased the expression of FPN1. This difference may be associated with cell type. For example, intestinal epithelial cells and placental cells are responsible for the transportation of exogenous iron into plasma; thus, the molecular mechanisms underlying iron metabolism may be different from those of other cells. In addition, Marro *et al* (34) reported that FAC added *in vitro* did not affect the expression of FPN1 in the mouse macrophage cell line RAW 264.7. However, the results of the present study are not consistent with these observations, which may be explained by differences in the concentrations of FAC. In the previous study, 2 μmol/l FAC was applied; this concentration was 10–100 fold lower than that applied in the present study. The normal serum iron concentration in the human body is 12.5–30 μmol/l, and in the case of an iron overload, the concentration can increase to >50 μmol/l. Therefore, FAC at a higher concentration may simulate iron overload *in vivo* more closely.

In conclusion, excessive iron increased the expression of FPN1 in osteoblasts, while iron deficiency decreased the expression of FPN1 in osteoblasts. The regulation of FPN1 in osteoblasts by iron may occur at transcriptional and translational levels. These observations indicate that FPN1 plays an important role in iron metabolism in osteoblasts.
